# No Detectable Fertility Benefit from a Single Additional Mating in Wild Stalk-Eyed Flies

**DOI:** 10.1371/journal.pone.0014309

**Published:** 2010-12-13

**Authors:** Elisabeth Harley, Kevin Fowler, Samuel Cotton

**Affiliations:** Research Department of Genetics, Evolution & Environment, University College London, London, United Kingdom; University of Oxford, United Kingdom

## Abstract

**Background:**

Multiple mating by female insects is widespread, and the explanation(s) for repeated mating by females has been the subject of much discussion. Females may profit from mating multiply through direct material benefits that increase their own reproductive output, or indirect genetic benefits that increase offspring fitness. One particular direct benefit that has attracted significant attention is that of fertility assurance, as females often need to mate multiply to achieve high fertility. This hypothesis has never been tested in a wild insect population.

**Methodology/Principal Findings:**

Female Malaysian stalk-eyed flies (*Teleopsis dalmanni*) mate repeatedly during their lifetime, and have been shown to be sperm limited under both laboratory and field conditions. Here we ask whether receiving an additional mating alleviates sperm limitation in wild females. In our experiment one group of females received a single additional mating, while a control group received an interrupted, and therefore unsuccessful, mating. Females that received an additional mating did not lay more fertilised eggs in total, nor did they lay proportionately more fertilised eggs. Female fertility declined significantly through time, demonstrating that females were sperm limited. However, receipt of an additional mating did not significantly alter the rate of this decline.

**Conclusions/Significance:**

Our data suggest that the fertility consequences of a single additional mating were small. We discuss this effect (or lack thereof), and suggest that it is likely to be attributed to small ejaculate size, a high proportion of failed copulations, and the presence of X-linked meiotic drive in this species.

## Introduction

Multiple mating in insects is a widespread phenomenon that has attracted much attention and for which many explanations have been proposed [1]; [2]; [3]; [4]; [5]; [6]; [7]. From a classical perspective, males are expected to increase their fitness by mating multiply with many females, while females are assumed to require only one or a few matings to maximise their fertility [3]; [8]. However, multiple mating by females is characteristic in many species, despite the potentially large costs that females incur from doing so [1]. As a result much work has focussed on female re-mating to seek explanations for this apparently paradoxical behaviour [2]; [3].

Multiple mating by females is likely to be costly as a result of ecological risks [1]; [9]; [10], costs derived from the act of mating itself [11]; [12]; [13], and even increased rates of polyspermy or the expression of adaptations for sperm competition that reduce fertility [14]. Advantages of mating multiply are usually classed as either direct or indirect (genetic) benefits [3]; [4]. Females may derive direct benefits from multiple copulations when males provide nuptial gifts that enhance female fitness directly [1]. Alternatively, if males transfer insufficient sperm in a single ejaculate to fertilize all of a female's eggs [1]; [2], or if fertility is limited by substances other than sperm that are also transferred during mating [15]; [16], then multiple mating increases female fitness directly by assuring long-term fertility [2]; [3]. Indirect benefits to females may also ensue if multiple mating results in the production of genetically superior offspring, for example if sperm competition engenders fertilisation of eggs by genetically superior or more compatible males [17]; [18].

There is good evidence from many insect species that multiply-mated females have higher fertility than those that have only mated once [19]; [20]. Such comparisons clearly demonstrate that multiple mating confers fertility advantages. However, why do females who have already mated multiply continue to do so? It is currently unclear whether additional matings by females that have already mated multiply also increase fertility; for example, re-mating frequency had inconsistent effects on fertility in dung flies [21], leaf beetles [22] and field crickets [23]. One might expect any immediate fertility benefit of an additional mating to decline with the frequency of female re-mating [23], if fertility approaches a maximum or if a female's sperm storage reaches capacity. Nonetheless, re-mating may still be important in the longer term if it maintains high fertility by replenishing used, lost, or dead sperm. Under such circumstances, correlations between mating frequency and fertility will be relatively uninformative about the adaptive value of female re-mating, as they reveal little about sperm depletion over time in females. To detect this ‘hidden’ fertility advantage of multiple mating requires experimental intervention that allows detection of temporal declines in fertility when females are denied additional matings [24].

The majority of investigations into multiple mating and its associated benefits in insects have been conducted under laboratory conditions [3]. Laboratory studies allow powerful, systematic and controlled investigations of mating behaviour and its consequences. However, laboratory conditions are also largely uniform and potentially unrepresentative of the natural environment under which mating traits originally evolved. In order to fully understand the forces that shape female mating behaviour, we need to also address questions concerning the benefits of female remating under natural conditions. This study is the first to examine fertility benefits associated with female multiple mating in a wild insect population.

Here, we investigate whether females gain fertility benefits from additional mating in a wild population of the polygamous Malaysian stalk-eyed fly, *Teleopsis dalmanni* (Diptera, Diopsidae). Stalk-eyed flies are characterised by lateral extensions of the head capsule, on which their eyes are located. In *T. dalmanni* the distance between the eyes (eyespan) is a sexually dimorphic trait, with males having greatly exaggerated eyespans compared to females [25]. They form nocturnal lekking aggregations at dawn and dusk, during which copulations take place [26]; [27]. Fights between males for control of these aggregations are typically won by individuals with greater eyespan [27], and females prefer to roost and mate with large eyespan males [24]; [28]; [29]; [30].

Both sexes of *T. dalmanni* are highly promiscuous and mate at high frequencies [31]; [32]; [33]. Male mating rate is heritable [33], but there is no evidence that female mating rate is genetically correlated with that of males [34]. Females usually have low fertility, and continually mated females lay a higher percentage of fertile eggs than females mated three times or those mated only once (81%, 62% and 40% respectively, [19]). This suggests that females re-mate to obtain direct fertility benefits, at least in laboratory populations. There is no evidence for fertility advantages arising from polyandry, as distinct from multiple mating, in this species [19]. The act of mating *per se* does not appear to be particularly costly, in terms of lifespan and lifetime fecundity, in *T. dalmanni* [32]. However, multiple mating may incur other, ecological costs [1]; [9]; [10]. Low fertility in female *T. dalmanni* is likely the result of sperm-limitation [19]. Males transfer few sperm during a single copulation (∼ 65 [35]; ∼142 [36]), and spermatophores are small [37] and unlikely to provide females with non-sperm resource benefits. Low fertility and chronic sperm limitation has also been documented in a wild *T. dalmanni* population. In a recent field study, Cotton et al. [24] found that only around 55% of eggs laid by wild females were fertilised, and that fertility declined with time when females were denied access to males. This implies that females face sperm-limitation over both the short and long-term.

We used a wild *T. dalmanni* population to test whether non-virgin wild females that received a single mating had higher fertility than a group of wild females that received an interrupted and incomplete mating. This approach allowed us to examine the fertility benefits of performing an additional mating, in an *n*+1 versus *n* mating design, where *n* is the mating frequency of females prior to the start of the experiment. We define females in the interrupted (*n*) mating group as controls and those allowed an additional (*n*+1) mating as experimental females. Given the low levels of female fertility and severe sperm limitation observed previously in this wild population [24], we asked whether a single copulation confers a significant reproductive advantage to a female by a) increasing fertility, and b) slowing the rate at which fertility declines when a female is housed in isolation.

## Materials and Methods

Fieldwork was carried out in Ulu Gombak, Peninsular Malaysia (3°19′ N, 101°45′ E) during March and September 2009. Observations of females, conducted by E.H. and S.C., took place during dusk (1800 to 1930 hours) at three distinct lekking areas (LD, BW and UBW). These sites were located along two adjacent tributaries of the Gombak river that were within 100 metres of each other.

To estimate the effect of a single mating on female reproductive output, we experimentally manipulated matings between wild flies. To ensure that they were sexually mature and receptive, all focal females were chosen once they had begun copulation, defined as engagement of male and female genitalia. At this point they were randomly assigned to one of two groups. Mated (M) females (*n* = 43) were allowed to continue mating before being captured. Matings were classified as successful when copulation lasted >30s; this ensured that complete spermatophore transfer had occurred [38]; [39]. Interrupted mating (IM) (*n* = 44) females were separated from their mate and captured before 30s of copulation had elapsed. Matings were interrupted by using a pencil or paintbrush to gently separate the male and female. Un-manipulated females from the mated group, which copulated for <30s were reclassified into the interrupted female group. Interrupted copulations do not result in sperm transfer, although they may lead to the transfer of seminal fluid [40]. Females were captured from the leks by aspiration into a plastic bag, and transferred into individual 500 ml containers within one hour of capture. These containers were lined with a moist cotton pad and a tissue paper base, and females were fed every two days with pureed banana.

Eggs laid on the tissue paper bases were collected from the containers every two days for 10 days following capture, and allowed to develop for a further five days in a Petri dish containing a moist cotton pad and pureed banana. Egg fertility was estimated by scoring hatching success under a light microscope at 10x magnification. Fertilised eggs that have hatched appear as empty chorion cases, while unfertilised eggs are full and show no signs of development. Sometimes fertilised eggs failed to hatch, but still showed signs of development (e.g. horizontal striations in the chorion and early mouthpart formation) [19]. These eggs were recorded as fertile.

Once egg collection was completed, females were killed and stored in ethanol. On return to the UK, females were measured for eyespan and thorax length using a monocular microscope and the image analysis programme ImageJ (Version 1.43e; National Institutes of Health, USA). Eyespan was defined as the distance between the outer tips of the eyes, and thorax length was measured along a midline from the base of the head to the joint between the metathoracic legs and the thorax [24]. Both measurements were made to an accuracy of 0.01 mm.

We evaluated the factors that affected female reproductive output using general linear models (GLMs). Female reproductive output was measured as number of eggs laid (fecundity) and number of eggs fertilised (absolute fertility). Since absolute fertility and fecundity were highly correlated (*F*_1,85_ = 1522.773, *p*<0.0001), we also estimated the relative number of eggs fertilised (relative fertility) by including fecundity as a covariate in GLMs explaining variation in absolute fertility. Females that failed to lay any eggs during the observation period (*n* = 8) were not included in the final models.

We found significant geographic variation in all aspects of female reproductive output (three sites: BW, UBW and LD, *n* = 7, 34 and 46 females respectively; all *F*_2,67_≥4.3526, all *p*≤0.0167), and as a result sample site was included in all models as a main effect. All site interactions were found to be non-significant so were not included in the models. We also investigated whether female morphology (eyespan and thorax length) had significant effects upon reproductive output.

We asked whether a single additional mating had any significant effects on female reproductive output by looking at treatment differences (i.e. *n*+1 matings in the M group versus *n* matings in the IM group) in fecundity, and absolute and relative fertility. We used data summed over the 10-day collection period to estimate overall reproductive output during the experiment. We also report the number of fertilised eggs as a percentage of total fecundity during the experiment for each group. Note that the sample site effect was not significant (*F*_2,64_ = 2.5372, *p* = 0.0870), and hence not included in the test of percentage fertility. Changes in the reproductive output of wild females during enforced time in captivity have previously been reported [24]. To determine whether fecundity and (relative and percentage) fertility changed over the captivity period (5×2-day egg collections), we included assay period as an ordinal factor (time in captivity) in the GLMs. Since eggs were collected from each female five times during the sample period, we included female identity as a random factor (shrunk by REML estimation) to account for non-independence of within-female measures.

All statistical analyses were performed using JMP software (version 5.0.1a, SAS Institute Inc.).

## Results

**Figure 1:**
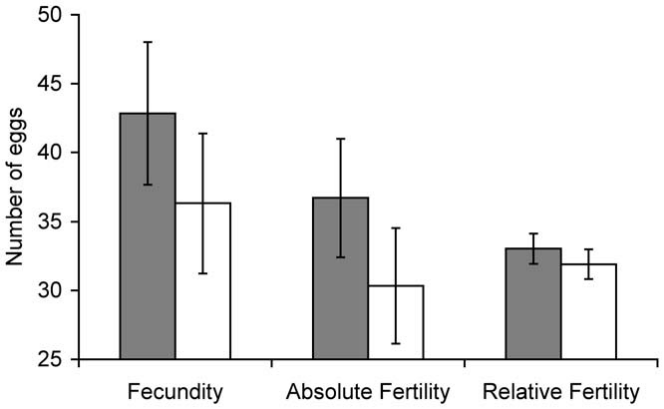
Fecundity, absolute fertility and relative fertility, summed over the 10-day observation period, for females that received either a single additional mating (shaded bars) or an interrupted mating (open bars). Data displayed as least squares means ± SE.

We found no significant difference between mated and interrupted females in terms of their total fecundity (*F*_1,63_ = 0.0466, *p* = 0.8298), absolute fertility (*F*_1,63_ = 0.1759, *p* = 0.6763) or relative fertility (*F*_1,62_ = 0.5762, *p* = 0.4507) over the 10 day sample period (Fig. 1). The percentage of fertile eggs in the mated group did not differ significantly from that in the interrupted group (mean%±SE: mated = 83.02±2.55, unmated = 78.09±2.75, *F*_1,65_ = 1.7297, *p* = 0.1931).

**Figure 2:**
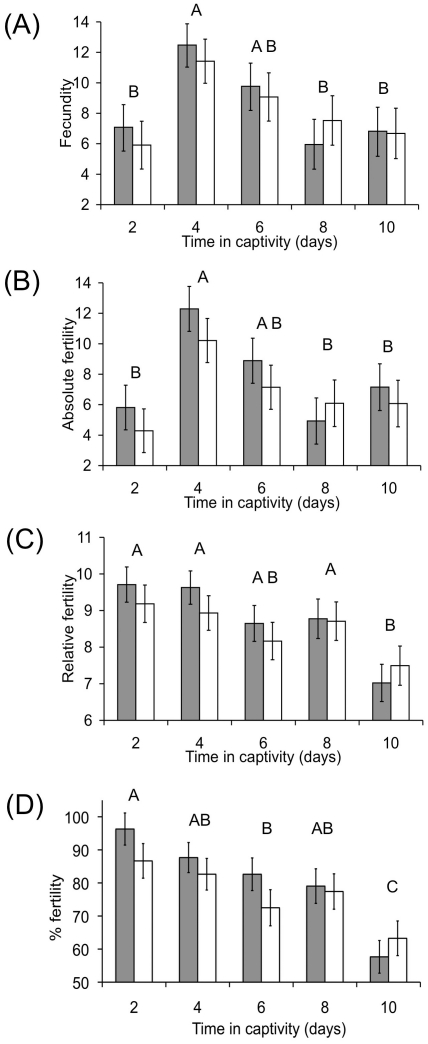
Changes in fecundity (A), absolute fertility (B) relative fertility (C) and percentage fertility (D) over time in captivity for females that received a single additional mating (shaded bars) or an interrupted mating (open bars). Time periods that are not connected by the same letter are significantly different (Tukey HSD comparison of pooled (mated plus interrupted) means). Data displayed as least squares means ± SE.

All aspects of female reproductive output varied significantly with time in captivity, but in different ways (Fig. 2). Both fecundity and absolute fertility showed significant peaks on day four of observation (Fig. 2A and 2B; fecundity: *F*_4,282_ = 7.5978, *p*<0.0001; absolute fertility: *F*_4,194_ = 5.9830, *p* = 0.0001). This effect is probably a short-term response to capture, and subsequent acclimatisation to captivity. In agreement with a previous study [24] we observed that relative fertility declined significantly with time (Fig. 2C; *F*_4,193_ = 6.9897, *p*<0.0001). We found the same pattern when we examined percentage fertility (Fig. 2D; *F*_4,193_ = 12.1636, *p*<0.0001). This is most likely the result of females being sperm limited after isolation from males [19]; [24]; [41].

If a single mating were able to alleviate sperm-limitation, we would expect differences between treatment groups; fertility in recently mated females should show less of a decline over time compared to that of interrupted females. However we did not observe this, as there was no significant interaction between treatment and time for both relative (*F*_4,188_ = 0.5580, *p* = 0.6934) and percentage fertility (*F*_4,188_ = 0.9161, *p* = 0.4557). Similarly, the treatment × time interactions were also non-significant for fecundity (*F*_4, 277_ = 0.3317, *p* = 0.8565) and absolute fertility (*F*_4,189_ = 0.2761, *p* = 0.8932), supporting the view that a single mating does not have an appreciable effect on female reproductive output (Fig. 2).

Neither female eyespan (fecundity: *F*_1,58_ = 1.2314, *p* = 0.2717; absolute fertility: *F*_1,58_ = 1.4236, *p* = 0.2377; relative fertility: *F*_1,57_ = 0.2065, *p* = 0.6513; percentage fertility: *F*_1,58_ = 0.0764, *p* = 0.7832) nor thorax length (fecundity: *F*_1,58_ = 0.8337, *p* = 0.3650; absolute fertility: *F*_1,58_ = 0.2266, *p* = 0.6359; relative fertility: *F*_1,57_ = 2.3604, *p* = 0.1300; percentage fertility: *F*_1,58_ = 0.0108, *p* = 0.9176) had significant effects upon female reproductive output.

## Discussion

One of the most compelling explanations for the occurrence of multiple mating in female insects is that they acquire direct fertility benefits from such behaviour [2]; [3]. Females often suffer from sperm-limitation, and there are numerous examples in which multiply mated females have higher fertility than once mated females [19]; [20]. However, evidence for continued fertility benefits from additional matings by females that have already re-mated is equivocal [21]; [22]; [23]. A previous field study on the same Malaysian population of *T. dalmanni* assayed here showed that wild females had low fertility (∼55%) and were highly sperm limited [24]. Males transfer few sperm during copulation [35]; [36], and additional non-sperm direct benefits are unlikely as spermatophores are small [37]. Given these extreme attributes, we asked whether receiving an additional single mating could alleviate sperm-limitation and confer significant reproductive advantages upon wild *T. dalmanni* females?

We used an *n*+1 *versus n* mating design to evaluate the fertility benefits that arise from an additional mating, relative to the background level of mating in the population. All of the females analysed were fecund and observed to begin copulating under natural field conditions with their mate of choice, showing that they were both sexually mature and receptive to mating. Contrary to a previous, laboratory-based, study on the fertility benefits of multiple mating in *T. dalmanni* [19], we found no evidence for continued fertility benefits from additional mating in wild females, measured using absolute or relative values, or when expressed as a percentage of the total number of eggs laid. This was surprising, since around one fifth of eggs laid were infertile. We also observed a significant decrease in fertility during the time in captivity when females were unable to re-mate. This indicates that they suffered from sperm limitation [24]. However, the rate of this decline in fertility was unaffected by an additional mating, which implies that a single mating was unable to mitigate sperm limitation. Therefore, against a background of natural mating behaviour, we found no detectable fertility benefits associated with a single mating.

Variability in the background level of mating (*n*) in our experimental design could potentially have obscured any fertility effects caused by the additional, experimental, mating. However, females in each group were chosen at random from the population, so there is no reason to believe that *n* differed significantly between the mated (*n*+1) and interrupted mating (*n*) treatments. However, this assumption is hard to confirm. Nonetheless, this apparent shortcoming of our design has the advantage that it frames the effect of a single additional mating relative to the natural mating background. Thus the fertility benefit (or lack thereof) that we observed is a realistic estimate of that gained by the average female in the population.

Why did we not observe fertility benefits from a single additional mating, despite the presence of unfertilised eggs and high sperm-limitation? One possibility comes from the observation that male *T. dalmanni* transfer few sperm during a single mating [35]; [36], and around a third of matings do not result in sperm transfer at all, despite lasting for longer than 30 seconds [19]; [40]. A similarly high proportion of failed copulations has been reported in other insects that transfer sperm via spermatophores; for example, in a noctuid moth species 20% of copulations between virgins failed to transfer any sperm to the female's storage organs [42]. In stalk-eyed flies the proportion of copulations that fail to transfer sperm is currently unknown under field conditions. However, if the failure rate is similar to that observed in the laboratory [19], then it is perhaps unsurprising that no difference was detected in the fertility of females mated *n*+1 times relative to those that mated *n* times. Under such circumstances, females may mate at high frequencies [31]; [32]; [43] in order to accumulate fertility over many matings. Future work should explore the number of additional matings required to significantly elevate female fertility relative to the background level, for example in an *n+i versus n* mating design, where *i*>1.

Any effect on fertility of low sperm number and a high proportion of failed copulations may be further exacerbated by the presence of an X-linked meiotic drive element [44]. The element is reported to occur in around 13–17% of males derived from wild populations of *T. dalmanni*, and is also found in its sister species, *T. whitei* [44]. Meiotic drive disrupts spermatogenesis, impairing the elongation of Y-carrying sperm and thus reducing their ability to fertilise [45]; [46]; [47]. As a result, females mated to drive-carrying males suffer from impaired fertility [45]; [46]. In *T. whitei* the ejaculate of non-drive males can reduce the competitive ability of sperm from drive males [40]; [48]. If this is also the case in *T. dalmanni,* then females may mate multiply in order to counteract the detrimental effects associated with mating with drive-bearing males. Indeed, higher frequencies of female multiple mating have been observed in laboratory populations of both *T. dalmanni* and *T. whitei* where meiotic drive is also present [49].

In spite of the factors that may constrain fertility, females in our study nonetheless exhibited higher fertility (∼80%) than those in a previous study on the same population (∼55% [24]). This suggests that there is temporal variation in either mating rate, male fertility, or both. Since the fertility derived from a single additional mating might be expected to decrease as existing fertility approaches a maximum, or if a female's sperm storage reaches capacity, our lack of discovery of fertility benefits may reflect the (relatively) high overall fertility in the population. In addition, since fertility is correlated with female mating history and sperm-limitation [19], females with relatively empty sperm storage organs would be expected to gain greater fertility benefits from an additional mating than females whose storage organs are full. It would therefore be informative to quantify the fertility added by a single mating in other populations or at different times, in which the average background level of fertility is lower than that of the sample studied here.

Why do females continue to mate if they do not benefit from increased fertility? There are two, non-mutually exclusive, solutions. First, females may remate in order to accumulate fertility over many matings (see above). Second, there may be indirect benefits associated with multiple mating if females are polyandrous [4]; [50]. While polyandry is weakly associated with increased fertility [51] (although there is no evience for this in stalk-eyed flies [19]), it also allows sperm competition, which can promote fertilisation by genetically superior or more compatible males [6]; [17]; [18]; [52]; [53]. A suite of microsatellite markers is available for *T. dalmanni* [54] that allows paternity to be assigned to offspring [39]. So future studies should determine whether wild *T. dalmanni* females are indeed polyandrous and to what degree, or whether they simply mate repeatedly with the male who has control of their chosen lekking site. Controlled mating investigations under laboratory conditions should also explore whether the offspring of polyandrous females have greater (post-hatch) viability than those of monandrous females.

In conclusion, we were unable to detect any fertility benefits from a single additional mating in a wild population of promiscuous stalk-eyed flies. This effect is most likely attributable to small ejaculate size, the high proportion of failed copulations, and the presence of X-linked meiotic drive [19]; [45]; [46]. Other, indirect, benefits may also result from polyandry [4]; [18]; [53], but these hypotheses have yet to be tested in wild populations. Males appear to derive few fertility benefits from a single mating, as mating once with a female does not significantly increase reproductive output, although assignment of paternity to offspring is required to test this hypothesis sufficiently [53]; [55]. Nonetheless, our data suggest that the high mating rate observed in both sexes of this species may be an adaptation to accrue fertility over many matings.
